# Environmental Dust Particles Repelling from A Hydrophobic Surface under Electrostatic Influence

**DOI:** 10.1038/s41598-019-44992-9

**Published:** 2019-06-18

**Authors:** B. S. Yilbas, Hussain Al-Qahtani, Abdullah Al-Sharafi, Saeed Bahattab, Ghassan Hassan, N. Al-Aqeeli, M. Kassas

**Affiliations:** 1 Mechanical Engineering Department, King Fahd University of Petroleum and Minerals, Dhahran, Saudi Arabia; 2 Center of Excellence in Renewable Energy, King Fahd University of Petroleum & Minerals, Dhahran, Saudi Arabia; 3 Electrical Engineering Department, King Fahd University of Petroleum & Minerals, Dhahran, Saudi Arabia; 4 Senior Researcher, K.A.CARE Energy Research & Innovation Center, Dhahran, Saudi Arabia; 5 Researcher at K.A.CARE Energy Research, Innovation Center at Dhahran, Dhahran, Saudi Arabia

**Keywords:** Energy infrastructure, Mechanical engineering, Materials science

## Abstract

Environmental dust particles repelling from a hydrophobic surface under the electrostatic influence are considered and the dynamics of the dust particles are analyzed incorporating the high speed camera. The velocity of the repelled dust particles are formulated using the force balance incorporating the forces associated with the electrostatic repulsion, particle adhesion, particle drag, and the inflight particles interaction under the charge influence. The functionalized silica particles are deposited on the glass surface towards achieving a hydrophobic wetting state on the surface. An electronic circuitry is designed and built while generating the electrostatic effect, in the pulse form, on the dust particles located on the surface of the hydrophobic plate. Findings revealed that functionalized silica particles deposited surface results in hydrophobic wetting state with contact angle in the order of 158° ± 2° and contact angle hysteresis of 2° ± 1°. The electrostatic impulsive force generated on the plate surface enables to repel most of the sizes of the dust particles; however, some of the small dust particles remain as the residues on the surface after the electrostatic influence. The dust particle velocity predicted from the analytical formulation agrees with that obtained from the high speed camera data. The pinning force of the small size particles (0.6 µm≤), due to adhesion on the surface, is found to be larger than the average size particles (∼1.2 µm), which in turn, suppresses these particles repelling from the surface under the electrostatic influence. The residues of the dust particles on the as received glass surface after dust repelling are more than those residues on the hydrophobic surface. This behavior is associated with the dust particles adhesion on the surface. Consequently, hydrophobic wetting state on the plate surface improves the dust particle repelling from the surface.

## Introduction

Settlement of the environmental dust on surfaces is one of the major issues related to efficient operation of energy harvesting devices. This is because of the modification of the optical characteristics of surfaces by the dust particles; in which case, optical absorption, transmittance and reflectance of the surface change considerably. Climate change enhances the frequency and strength of the dust storms around the Globe, which becomes one of the important and frequent concerns of the designers and operators of the energy harvesting devices. Dust accumulation on active surfaces of the energy harvesting devices significantly reduces the device performance in terms of efficiency and output power^1^. The dust particles are consisted of various elements and sizes, and some of the dust particles can possess ionic charges while giving rise to the particle clustering in the dust layer. The ionic charge on the particles influences the adhesion of the clustered dust particles on the solid surfaces. Hence, the efforts required for the dust particles removal from the surfaces become excessively large. In humid air ambient conditions, water condensation on the dust particles can possibly dissolve the alkaline and alkaline earth metal compounds of the dust particles while forming a chemically active liquid solution around the particles^2^. The liquid solution has a basic nature (pH ≥ 7.8) and flows onto the solid surface under the gravitational potential. The liquid solution gives rise to damages on the solid surface via erosion and corrosion^2,3^; in which case, some of damage sites become permanent while modifying the texture, free energy, and optical characteristics of the surface. In addition, crystals are formed on the surface upon drying the film of the liquid solution^4^. The crystal structures strongly adhere to the solid surface and form intermediate layer between the dust particles and the solid surface. This behavior, in turn, enhances the dust particles adhesion on the surface; consequently, the efforts required for removing these particles from the surface are multiplied by many folds^5^. In order to minimize the film formation by the liquid solution on the solid surfaces, the wetting state of the surface can be changed to hydrophobic state. In this case, the spreading coefficient reduces below zero and the liquid solution forms locally dispersed islands of small liquid drops on the solid surfaces. Hence, the hydrophobic wetting state prevents film formation on the surface by the liquid solution^6^. In addition, hydrophobic surfaces lower the dust particle adhesion on the surface because of the low interfacial energy between the dust particles and the solid surface. Several techniques are introduced to remove the dust particles from the solid surfaces prior to wetting by the water condensate. One of the promising methods is to introduce repetitive electrostatic forces on the surface towards repelling the dust particles. The initial clustering of the dust particles on the solid surface, because of the ionic charges, can influence the electrostatic forces required for the removal of the dust particles from the surface. Consequently, investigation of environmental dust particles removal form the solid surfaces via applying repetitive external electrostatic forces becomes essential.

The environmental dust particles characteristics and their removal removal from the solid surfaces are examined previously^7–10^. Several methods are introduced, including droplet rolling on inclined hydrophobic surfaces^7^, mechanical brushing of surfaces^8^, air jet blowing on surfaces^9^, electrostatic repelling^10^, etc. In general, the external influence is generated for the removal of the dust particles from the hydrophobic surfaces. However, the wetting state of the surface may become a major concern for the dust particles removal from the solid surfaces through applying the electrostatic forces. The application of the electrostatic forces for the dust removal from the photovoltaic active surfaces was presented by Kawamoto and Guo^11^. The findings revealed that the energy required repelling the small size dust particles is higher than that of the large size particles. However, applying the high voltage enhances the dust particles repelling and improves the surface cleaning efficiency. The electrostatic dust removal from the biomass flue gas was investigated by Cui *et al*.^12^. They showed that the cleaning efficiency of the dust particles improved when the dust particles were located in T-shape on the surface and increasing applied voltage enhanced the dust repelling from the surface. The importance of electrostatic charge on the repelling of the dust particles from the solid was studied by Sayyah *et al*.^13^. They indicated that the ratio of electrostatic charge over the dust particles mass was influenced by the electric field intensity; in which case, increasing electric field intensity improved the amount of dust particles repelled from the solid surface. The electrostatic traveling wave and the dust removal from surfaces are studied by Kawamoto and Hashime^14^. The findings reveal that low frequency of surface vibration improved the dust repelling from the surface under the applied electrostatic forces. The surface topography and the dust particles transport under electrostatic effects were examined by Poppe *et al*.^15^. They adopted the particle-in-cell model to investigate the influence of surface topography on the transport characteristics of the dust particles. The dust particles behavior and their separation in electrostatic field were studied by Hofer and Wolter^16^. They demonstrated that the dust mixture displayed precipitation properties, which were associated with the mineralogical structures of the dusts. The mechanical behavior of un-compacted dust aggregates was investigated by Pontius and Snyder^17^. The mechanical response of the dust aggregates demonstrated the complicated behavior, which was related to the heterogeneity of inter-particle forces due to varying sizes of the dust particles. The mechanics of charged dust particles in electric field were examined by Tedjojuwono *et al*.^18^. The dynamic motion of the dust particles was dependent on the strength of the applied voltage, which was more pronounced for the small dust particles. The influence of the electrostatic forces on the motion of charged particles was studied by Jianan *et al*.^19^. They showed that the motion of the charged dust particles was influenced by the electrostatic repulsion among the particles and the size of the charged dust layer formed on the charged surface.

Although mechanics of the dust particles in the electrostatic field were investigated previously^10–19^, the general focus was to examine the particle electrostatic interactions in the charged field. The dynamics of the dust particles repelled from the hydrophilic and hydrophobic surfaces under repetitive charges are left for future study. The adhesion force remains large in between the dust particles and the glass surface because of the large interfacial energies and the tangential force required to remove the dust particles from the surface is significantly large. As the wetting state of the glass surface is changed from hydrophilic to hydrophobic, the interfacial energy between the dust particles and the hydrophobic surfaces becomes low because of the low free energy of the hydrophobic surface. Hence, adhesion force between the dust particles and the hydrophobic surface surfaces becomes low. Therefore, the dust particle dynamics on the hydrophobic surface is expected to be different than those on the hydrophilic surfaces when the electrostatic repulsive force is applied. Consequently, in the present study, the characteristics of the environmental dust particles and the texture feature of the hydrophobic surface are examined. The environmental dust particles dynamics under the repetitive electrostatic forces are investigated in relation to self-cleaning applications of the hydrophilic and hydrophobic glass surfaces. An electrostatic plate and accompanying electronic circuit are designed and manufactured for dynamic testing of the environmental dust particles. The behavior of the dust particles repelled from the surface under the electrostatic force is formulated and findings are compared to that obtained from experiment. High speed camera is used to monitor the dust particles motion in air when they are repelled from the hydrophobic and hydrophilic surfaces.

## Experimental

The glass samples with 30 mm × 50 mm × 0.2 mm (width × length × thickness dimensions were used in the experiments. The composition of the glass samples is 76.5% SiO_2_, 9.9% CaO, 1.2 MgO and 12.4% Na_2_O. Prior to the silica particles deposition, the glass surfaces were cleaned in a piranha solution. The nano-size silica particles were formed through the synthesizing process, which involved with tetraethyl orthosilicate (TEOS), 3-aminopropyltrimethoxysilane (AMPTS) and isobuthytrimethoxysilane (OTES), ethanol, and ammonium hydroxide. The synthesizing process was presented earlier and the processing particulars could be obtained from the work reported^20,21^. The solution consisting of the synthesized silica particles was mixed with the modifier silane molecules 3:4 molar ratio to functionalize the silica particles. The resulting solution was stirred for 15 hours at room temperature, later centrifuged, and washed with ethanol to remove the reactants in the mixture. The solvent casting technique was used coating the glass surface by functionalized particles. The deep coating technique was utilized incorporating the coating unit (Chemat Scientific KW 4AH by Chemat Technology Inc.). The coating chamber was spanned at a constant speed for ten minutes and the samples were pulled up from the coating chamber at a constant speed (0.1 mm/s). The surface was, then, vacuum dried ensuring all the solvents were removed from the surface. The treated surfaces were characterized assessing the wetting state and texture morphology. The functionalized silica particles deposition on the glass surface resulted in uniform wetting state with the droplet contact angle of 158° ± 2° and the hysteresis of 2° ± 1°.

The environmental dusts were collected using the soft brushes from the photovoltaic panel surfaces in the local area of Dammam in Kingdom of Saudi Arabia. The dust particles collected were stored in the sealed container prior to the experiments. The dust particles were analyzed in terms of the size, shape, elemental composition, and adhesion on the surface. The characterization tools include scanning electron microscope (SEM by JEOL 6460), energy dispersion spectrometry (EDS by JEOL 6460), and X-ray diffraction (XRD by Bruker D8 Advanced with Cu-K_α_ radiation source) and atomic force microscope (5100 AFM/SPM by Agilent). AFM tip was made from silicon nitride and the tip radius was r = 20–60 nm. The dust adhesion tests, in terms of the tangential measurements, was carried out in line with the previous study^22^ incorporating the friction mode of AFM tip.

The adhesion of the coating, produced by the deposition of the functionalized silica particles, on the glass surface was evaluated using the linear micro-scratch tester (MCTX-S/N: 01-04300). The contact and the end loads of the micro-scratch tester were maintained at 0.03 N and 2.5 N, respectively while the scanning speed was kept at 5 mm/min with the loading rate of 0.01 N/s.

**Figure 1:**
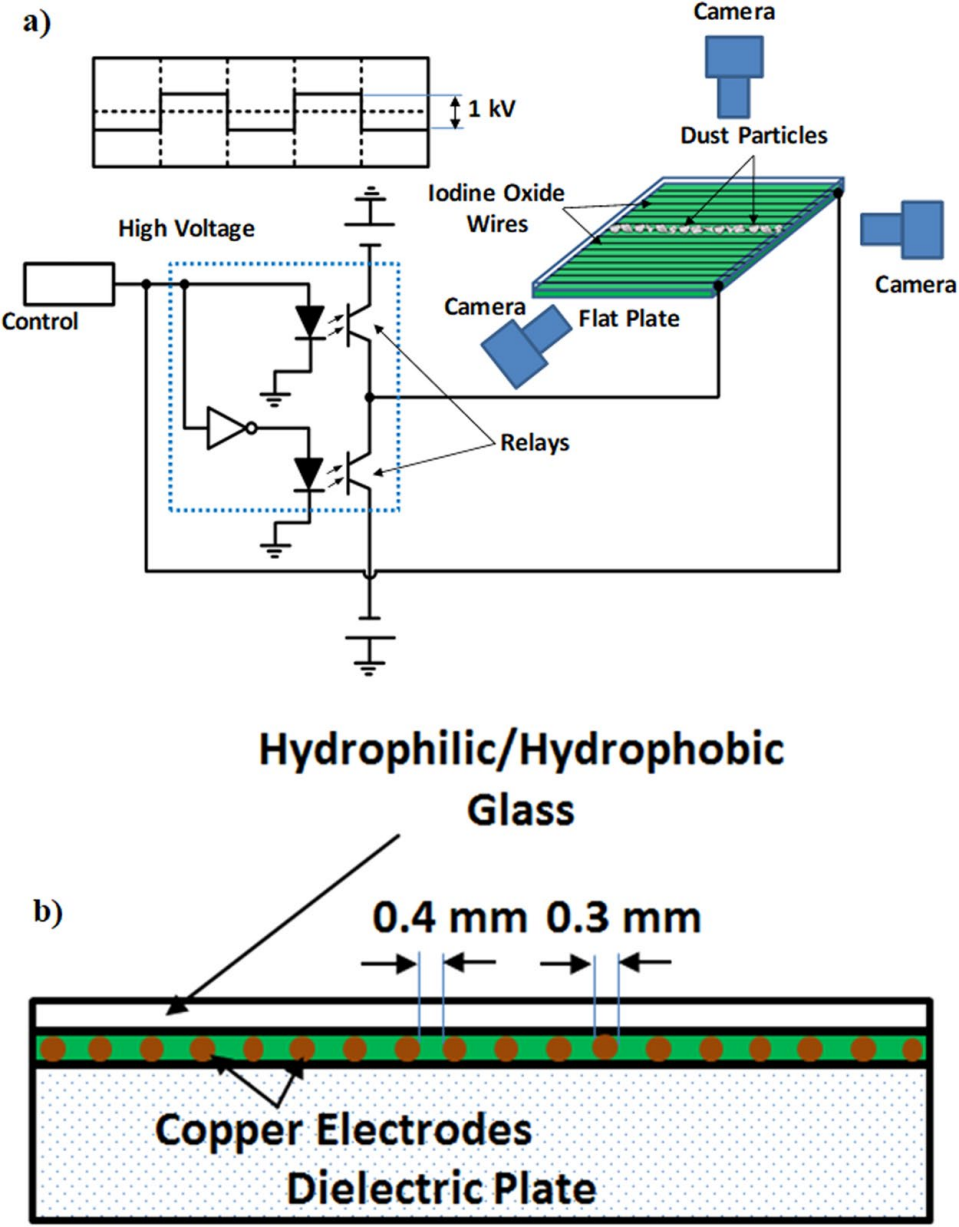
A schematic view of dust repelling system and flat plate: (**a**) dust repelling system and cameras, and (**b**) flat plate with electrodes.

An electronic circuit and copper electrodes with 0.3 mm diameter and 100 mm length, which was imbedded in a borosilicate glass, were used to generate electrostatic impulse on the plate surface. Figure 1a shows the schematic view of the electronic circuit and the plate while Fig. 1b depicts the size of the plate arrangement. In line with the previous study^23^, MOS relays (Panasonic, AQV258) were used for the high voltage pulse regulation and the relays were controlled by the electronic circuit to obtain the desired high voltage pulse form. In this case, amplifiers (HVNT-1P-5 and HVBT-1N-5) were used generating the high voltage pulse with 1 KV and 0.5 mA output and the length of the high voltage pulse was set at 0.4 s.

**Figure 2:**
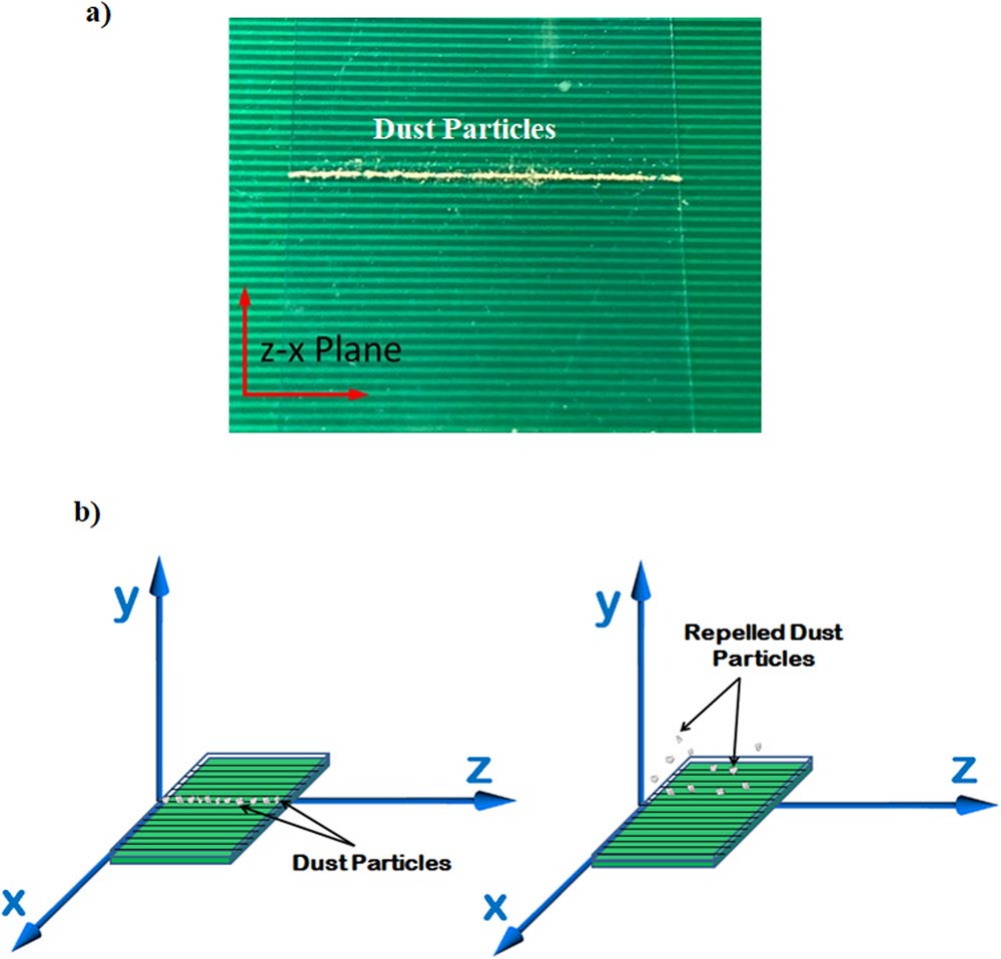
Optical image of dust particles on flat plate and coordinate system before and after dust particles repelling: (**a**) dust particles located along the electrode axis, and (**b**) location and coordinate system before and after dust particles repelling.

A high speed camera (Dantec Dynamics SpeedSense 9040) was used to monitor and record the trajectory of the dust repelled from the surface under the electrostatic influence. Three cameras were situated to observe the dust particle motion in 3-dimensions. A schematic view of the camera settings are shown in Fig. 1a. Figure 2 shows the optical image of the plate and the dust particles deposited along the copper electrode axis. The data recorded were analyzed via tracker program to obtain the velocity and acceleration components of the particle along x, y, and z-axes (Fig. 2). In order to trace the particle repelled from the surface, the particle size about 10 µm was selected.

## Results and Discussion

Dynamics of the dust particles repelled from the hydrophilic and the hydrophobic surfaces are examined. The dust particle velocity is formulated incorporating the affecting forces on the dust articles and the velocity predictions are compared with those obtained from the high speed camera data. The dust particles residues on both hydrophilic and hydrophobic surfaces are characterized using the analytical tools. The adhesion force for the dust particle on both surfaces is estimated from the atomic force microscopy data, and later, the findings are incorporated in the velocity formulations.

### Surface analysis and dust particles characteristics

**Figure 3:**
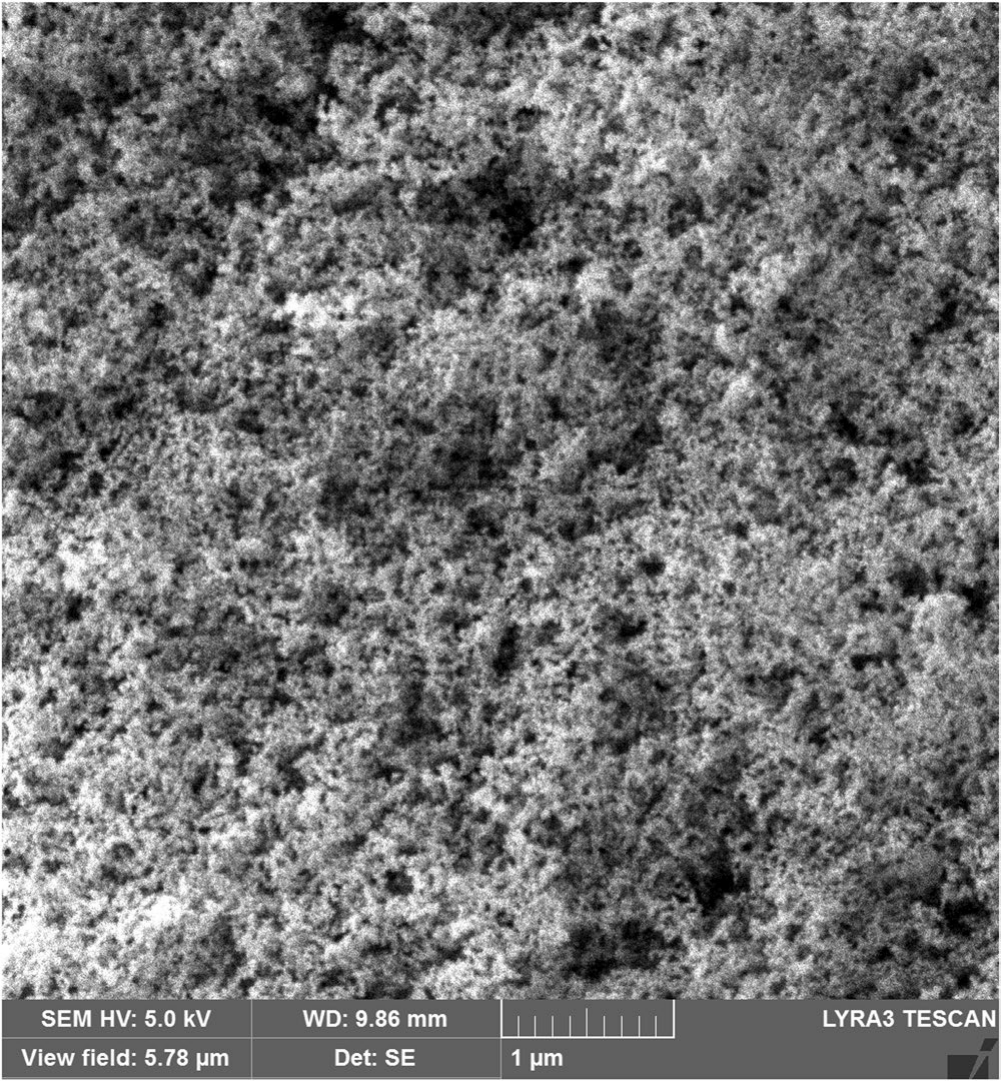
SEM micrograph of functionalized silica particles deposited surface.

**Figure 4:**
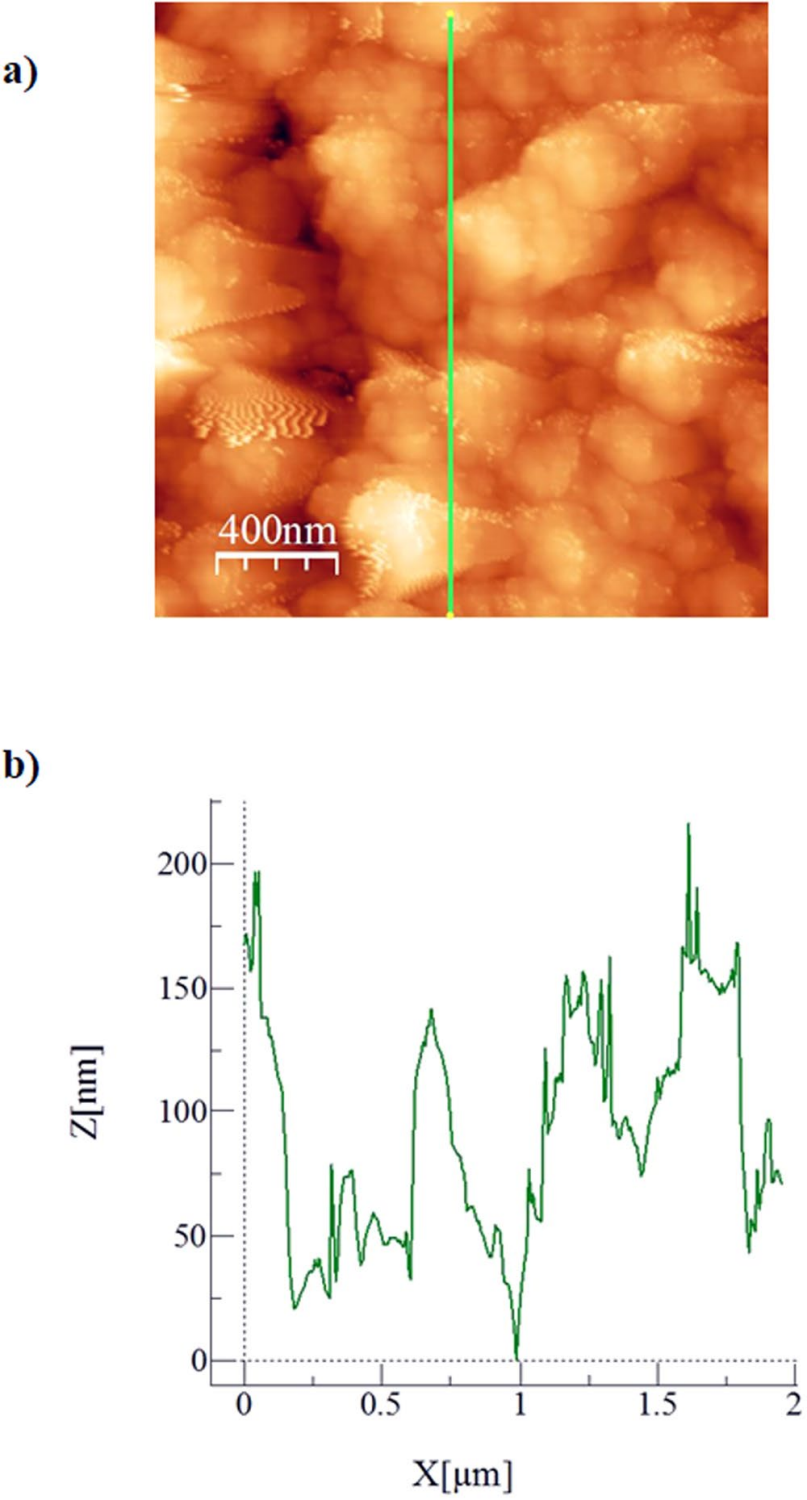
Atomic force microscopy image of functionalized silica particles deposited surface and line scan: (**a**) top view of functionalized silica particles deposited surface (green line shows the line scanning direction), and (**b**) line scan along the surface.

**Figure 5:**
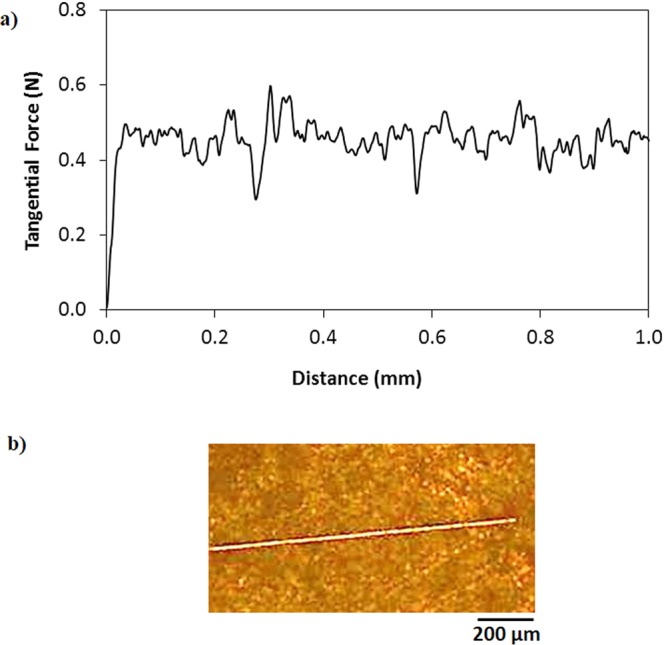
Tangential force and scratch mark obtained from micro-tribometer for functionalized silica particles deposited surface: (**a**) tangential force required removing the silica particles from the surface, and (**b**) indentation mark on the surface during scratch testing.

**Figure 6:**
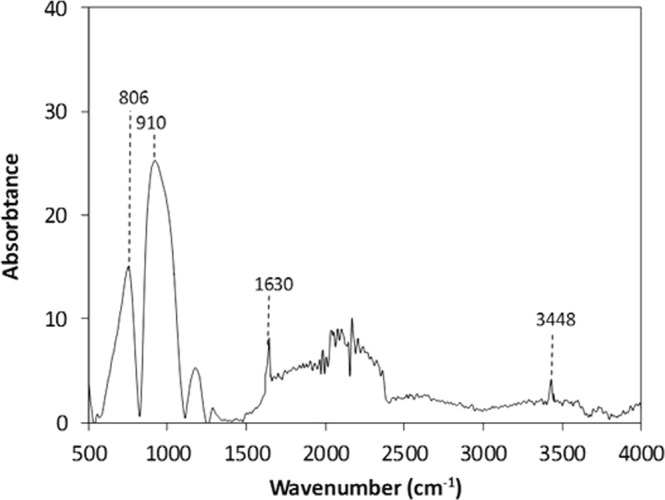
FTIR data for functionalized silica particles deposited surface.

Figure 3 shows SEM micrographs of silicon nano-size particles deposited surface. The particles are agglomerated on the surface forming cluster-like structures. The nano-size silica particles agglomeration is attributed to the silane used as modifier which triggers the side reactions leading the condensation on the silica surface. This contributes to the agglomeration of the particles^24^. Some pores-like structures are formed around the cluster-like structures. However, they are locally scattered in the irregular manner. The formation of the pores-like structures is mainly associated with the clustering of nano-size functionalized silica particles; in which, clusters particles adhere closely each other while forming the some small gaps on the surface. These gaps appear as the pores-like texture on the surface. In addition, the pores-like structures are not connected to each other rather forming individual gaps around the clustered nano-size particles. Figure 4a shows atomic force microscopy 2-dimensional image of the nano-size silica particles deposited surface while Fig. 4b shows the line scan along the surface, which is obtained from the atomic force microscopy. The presence of agglomerated silica particles on the surface gives rise to wavy texture profile with low amplitudes (heights). The pores-like texture alters the wavy-like profiles and causes large variation in the texture profile. This appears as deep cavity-like profile in Fig. 4b. The average surface roughness is in the order of 120 nm. In order to assess the adhesion between the nano-size functionalized silica particles coating and the glass surface, scratch tests are carried out using the micro-tribometer. Figure 5a shows the tangential force required removing the silica particles from the surfaced while Fig. 5b shows the indentation mark left on the surface after the scratch tests. The tangential force does not vary significantly along the surface, which demonstrates almost uniform adhesion of the nano-size silica particles on the glass surface. However, some small oscillations in the tangential force curve are associated with the pores-like structures, where the agglomeration of the nano-size silica particles is less. The scratch width remains almost same along the scratch length (Fig. 5b), which also indicates the uniform deposition and attachment of the nano-size silica particles on the surface. Figure 6 shows FTIR data obtained for the silica nano-size particles deposited surface. The peak occurring on 806 cm^−1^ band corresponds to O-Si-O bending vibration^25^. In addition, the peak observed on 1630 cm^−1^ band represents the stretching vibration of Si-OH groups in the functionalized silica particles^25,26^. The hydroxyl group (Si-OH group) indicates the presence of bounded water. The peak at 3448 cm^−1^ is related to the stretching vibrations O-H on silica surface while demonstrating the surfaces are not fully covered with the grafted groups. The addition of tetraethylorthosilicate (TEOS) during the functionalizing of synthesized silica particles gives rise to formation of soluble silicates and OH^−^ ions triggers the hydrolysis of TEOS,^26^. Stretching of CH_2_ peaks occurs on 2852 cm^−1^ band, which is related to the treatment of silica nano-size particles with octadecyltrichlorosilane (OTES) during the functionalizing process^27^.

The surface free energy of the silica nano-particles deposited surface is assessed using the droplet method^28^. Hence, the contact angle is measurements are carried out for water, glycerol, and diiodomethane in accordance with the early study^28^. The arrangements of the surface free energy estimations are provided in the Supplementary Material S1. Table 2 gives the data used in the surface energy assessments. The surface energy determined for the functionalized nano-size silica particles deposited surface is about 38.24 mN/m, which is lower than that reported for CVD plasma enhanced tetraethylorthosilicate (PE-TEOS) coated surface (42.12 mN/m)^29^. The water droplet contact angle for the silica nano-particles deposited surface is 158° ± 2° with hysteresis of 2° ± 1°. The deposited surface demonstrates the superhydrophobic wetting state having extremely low contact angle hysteresis. The measurement of surface energy of the deposited surface is repeated 10 different locations on the surface. The findings reveal that the variation of the values of the free energy over the surface is negligibly small, i.e. the surface has almost uniform free energy with a significantly small variation. Hence, the functionalized nano-size silica particles coated surface has uniform wetting characteristics, which is associated with the uniformly distributed hierarchal texture structure and almost constant surface free energy.

**Table 1:** Elemental composition of dust (wt.%) determined by energy dispersive spectroscopy (EDS).

Particle Size	Si	Ca	Na	S	Mg	K	Fe	Cl	O
≥1.2 µm	12.4	8.5	3.2	2.1	2.5	1.2	1.1	0.9	Balance
≤0.8 µm	10.1	7.3	2.1	1.4	1.6	1.1	0.9	0.8	Balance

**Table 2:** Lifshitz-van der Walls components and electron-donor parameters used in the simulation^29,41^.

	γ_L_ (mJ/m^2^)	${{\boldsymbol{\gamma }}}_{{\boldsymbol{L}}}^{{\boldsymbol{L}}}$ (mJ/m^2^)	${{\boldsymbol{\gamma }}}_{{\boldsymbol{L}}}^{{\boldsymbol{+}}}$ (mJ/m^2^)	${{\boldsymbol{\gamma }}}_{{\boldsymbol{L}}}^{{\boldsymbol{-}}}$ (mJ/m^2^)
Water	72.8	21.8	25.5	25.5
Glycerol	64	34	3.92	57.4
Diiodomethane	50.8	50.8	0.72	0

**Figure 7:**
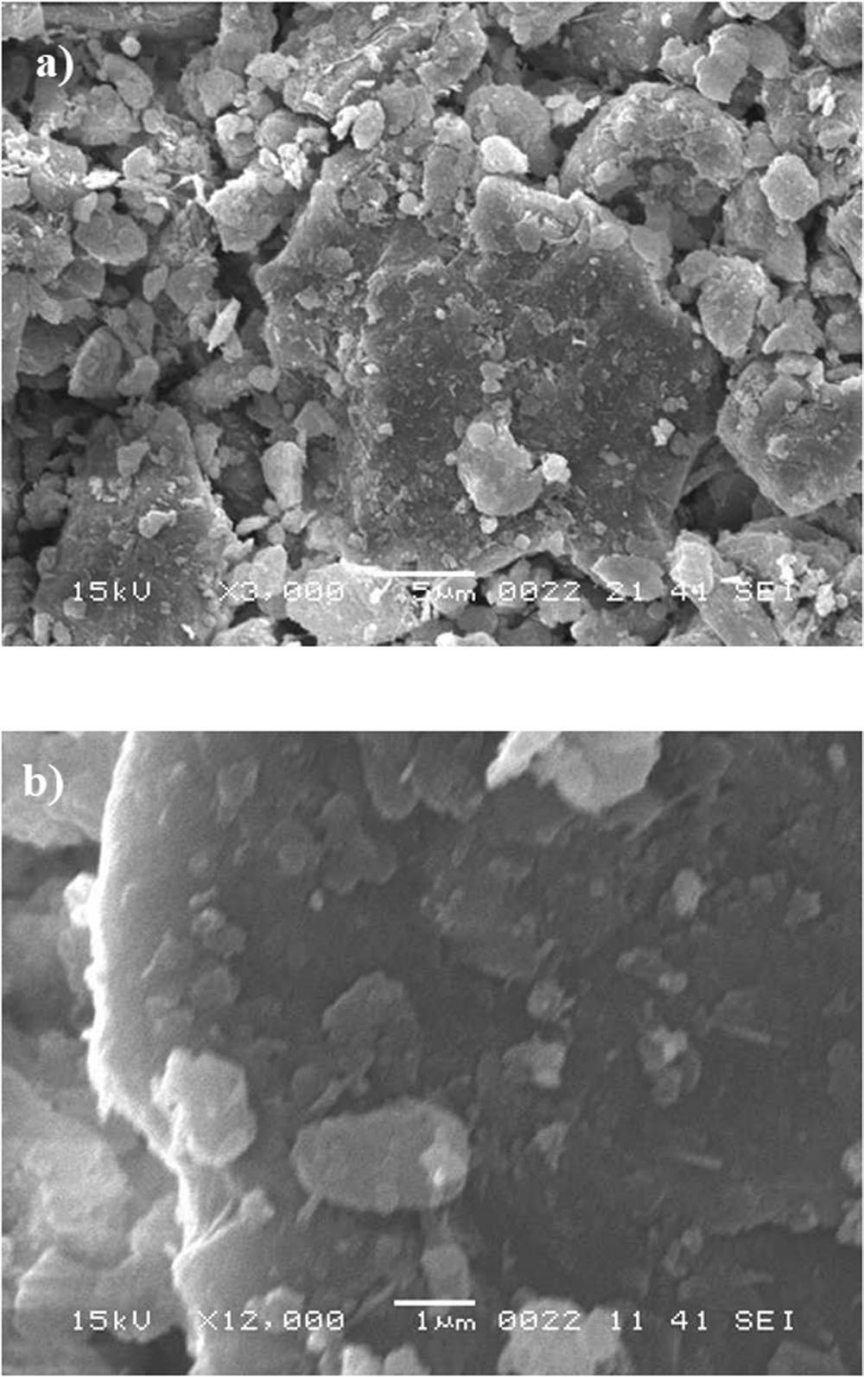
SEM micrographs of dust particles: (**a**) dust particles of various size and shapes, and (**b**) small dust particles attach at the large dust particle surface.

**Figure 8:**
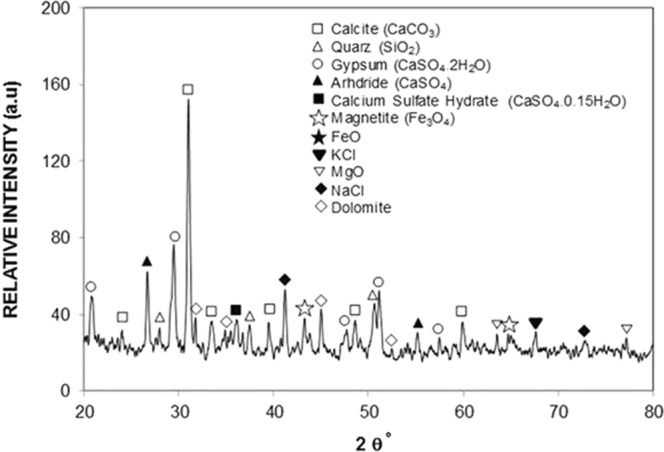
X-ray diffractogram of dust particles.

The dust particles have various shapes and sizes. This can be seen from Fig. 7a, in which SEM micrographs of the dust particles are shown. The small size dust particles attach at the surface of the large size dust particles (Fig. 7b). In addition, small size dust particles forms clusters around the large size particles. The small size dust particles suspend in air longer durations than the relatively larger size dust particles. Hence, it is possible that the interaction of solar irradiation with the small particles gives rise to bonding of some ionic components to the dust particles under the prolonged exposure in the near seashore areas (Arabian Peninsula). Nevertheless, the dust particles are weakly bonded and small dust particles partially disintegrate under the air blow. Energy dispersive spectroscopy (EDS) analysis for the elemental composition of the dust particles is provided in Table 1. The dust particles compose of various elements including Si, Ca, K, Na, Cl, S, O, Fe and Mg. The presence of the various elements in the dust particles is attributed to the local geological structure of the landscape. However, depending on the size of the dust particles, elemental composition varies slightly (Table 1). The concentrations of Na, K, Ca, O and Cl are slightly higher for the small size particles (<0.8 μm). Figure 8 depicts the X-ray diffractogram of the dust particles. The diffractogram demonstrates that various crystal compounds including alkaline and alkaline earth metal compounds are present in the dust particles. The iron peaks overlays with silica and the peak corresponds to the clay-aggregated hematite (Fe_2_O_3_). The sulfur peak is attributed to the anhydrite or gypsum (CaSO_4_) components in the dust. The dust particles have various shapes with non-regular characteristics. However, an attempt is made to group the dust particles in accordance with their shapes. In this case, the shape factor and the aspect ratio are introduced. The shape factor (*R*_*Shape*_), is associated with the ratio of dust particle perimeter over the total area of the dust particle, i.e. ${R}_{Shape}=\frac{{P}^{2}}{4\pi A}$, where *P* represents the dust particle perimeter and the *A* is the cross-sectional area of the dust particles. The aspect ratio (*A*_*Aspect*_) represents the dust particles approximate roundness. It is the ratio of the major-to-minor axes of an ellipsoid that is best fit to the particle geometry, i.e. ${A}_{Aspect}=\frac{\pi {L}_{Projction}^{2}}{4A}$, where *L*_*Projection*_ is the longest projection length of the dust particle and *A* is the cross-sectional area of the dust particles. The inverse relation is observed between the size of the dust particle and the aspect ratio; however, no simple relation is sorted between the dust size and the aspect ratio. As the shape factor increases, the particle aspect ratio reduces. This is particularly true the large size particles (>5 µm). The shape factor reduces to almost unity for the small size dust particles (<1.2 μm).

### Dynamics of dust particles repelling from surfaces under electrostatic influence

**Figure 9:**
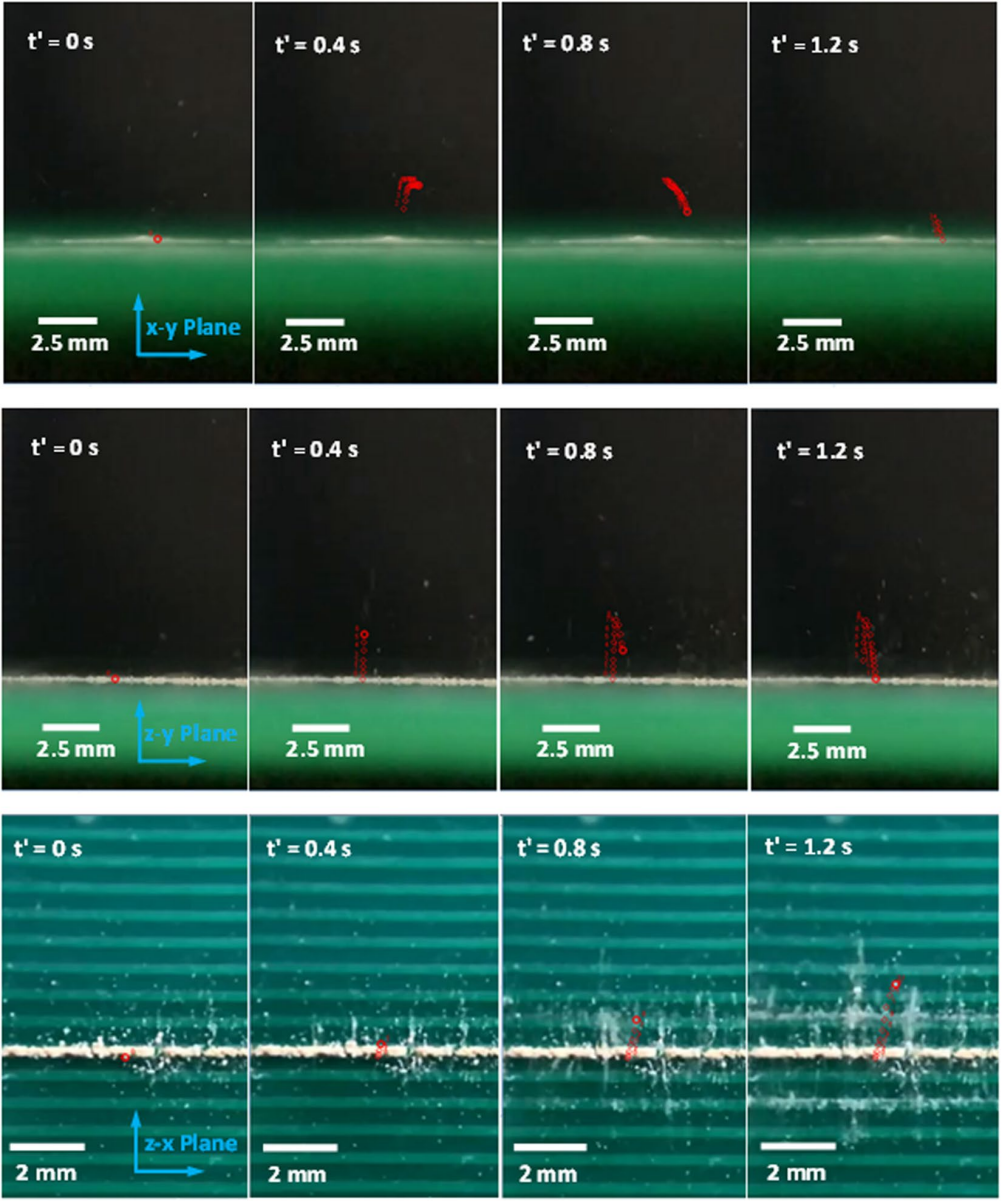
High speed camera images for the repelled dust particles (inflight) trajectories in air in y-x, z-y, and z-x plane.

In order to assess the particle trajectories when repelled from the surface by an electrostatic impulsive force, particles were traced with a high speed camera (Dantec Dynamics SpeedSense 9040). It should be noted that the size of the dust particle repelled is selected as about 10 µm in order to observe by the high speed camera with adopted micro-lens. Figure 9 shows the high speed camera image of one of the particles traced along x-y, y-z, and x-z planes at different times after the initiation of the impulsive electrostatic force. It should be noted that the particle size is about 10 µm and the particle traced is marked in red-circle for the particle visibility. The particle repelled demonstrates 3-dimensional trajectory behavior. The occurrence of the 3-dimensional trajectory is associated with one or all of the followings: (i) the location of the particle on the surface prior to repelling; in which case, the particle position reference to the electrostatic force center (electrode wire) remains critical, (ii) the particle shape is not regular and it is possible that the particle initial position on the surface (prior to repelling) is not parallel to the surface, i.e. the particle is located with a tilt angle on the surface, (iii) the retarding forces, such the drag and inter-particle charge forces acting on the particle surface (non-circular and complicated geometric feature), creates in balance on the gravitational pulling force while causing the modification of the particle trajectory, (iv) electrostatic changes of the dust particle changes because of different and non-uniform elemental composition of the dusts particles, (v) the size of the dust particles changes and the externally applied electrostatic influence for individual dust particles vary, and (vi) the adhesion forces in between the agglomerated dust particles on the surface influence the initial motion of the dust particle, which is repelled from the surface, and (v) mechanical interaction of dust particles onset of repelling from surface. In any case, the motion of the dust particles follows the curvilinear behavior at some elevation from the surface. This indicates that the interaction of the retarding forces with the gravitational force plays a major role on the trajectory of the particles repelled. Moreover, as the repelled dust particle is lifted further away from the surface, the electrostatic effect generated by the electrode, during 0.4 s pulse length of the high voltage excitation, influences the electrostatic force balance on the inflight dust particles while further altering the curvature of the dust particle trajectory. Consequently, the force balance among the retarding, gravitational pull, and particle inertia forces alters the curvature of the repelled particle trajectory, which is observed from Fig. 9 during the late flight timing of the repelled dust particle (=0.9 s). It should be noted that the high voltage pulse length is 0.4 s and the total duration of the dust particle including the repelling and the falling period is about 0.9 s. It should be noted that in high humid environments, water condensate can cause dissolution of alkaline (Na, K) and alkaline earth (Ca) metal compounds of the dust particles while forming a solution, which gives rise to the pore-filling minerals. Upon drying, the pore-filling minerals can form bridges between the dust particles while causing sedimentation^30^. Moreover, as the air humidity increases the capillary condensation of water vapor takes place on the contact surfaces of the particles. The capillary condensation of water along the interfacial surfaces between the dust particles influences the electrostatic forces^30^ while altering the cohesive forces towards cementation^31^. Hence, a care is taken to avoid the influence of capillary condensation at the dust interfaces. Consequently, the relative humidity of air is kept low (37%) during the experiments. Moreover, the microscopic examinations of the dust particles this situation is not observed.

**Figure 10:**
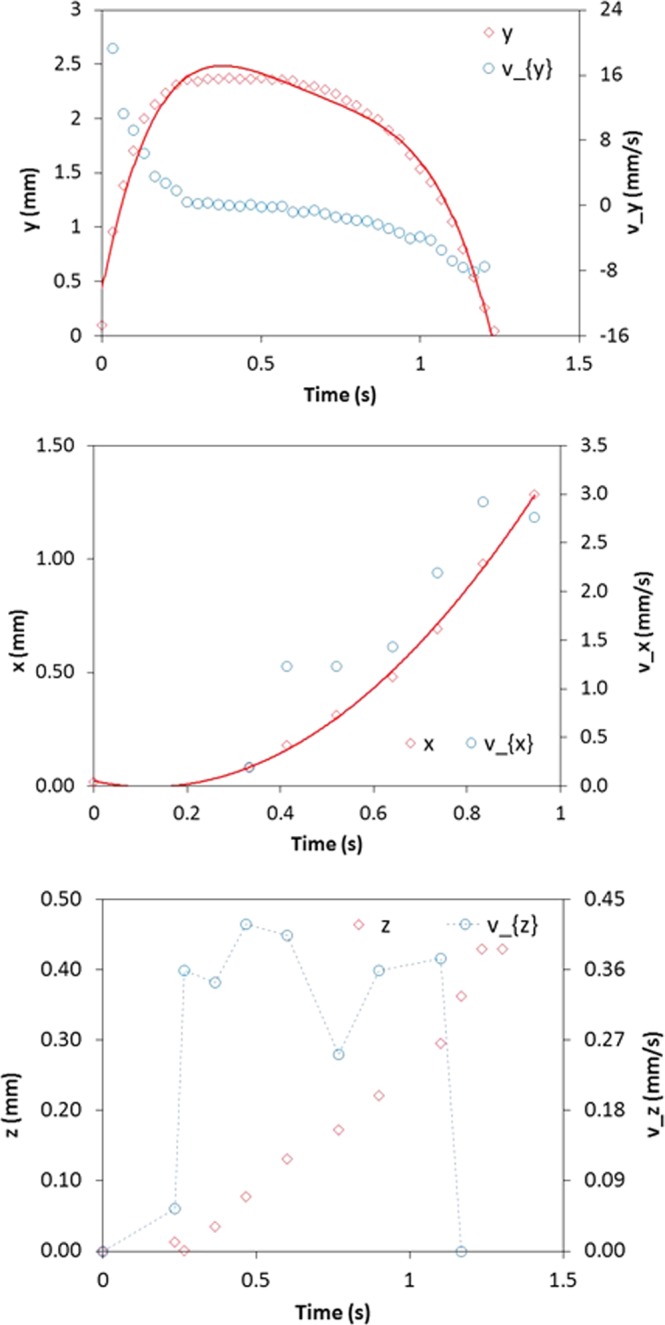
Dust particles velocity along, x, y, and z-axes together with the distance travelled by the repelled (inflight) dust particle in air. Negative velocity along y-axis depicts the landing particle on the surface after reaching its peak due to electrostatic influence.

**Figure 11:**
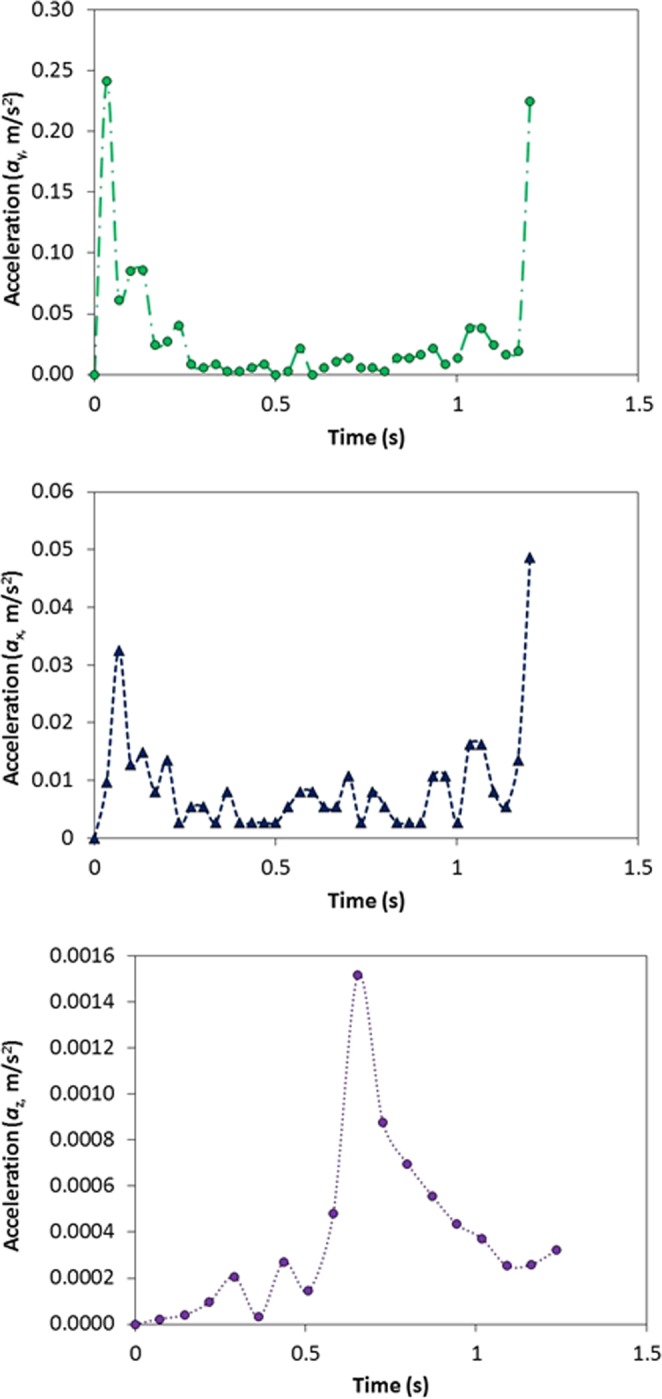
Dust particles acceleration along, x, y, and z-axes together with the distance travelled by the repelled (inflight) dust particle in air.

Figure 10 shows the velocity of the repelled particle along y, x, and z-axes, which are obtained from the high speed camera data. Since the dust particles are initially located along the electrode axis, where electrostatic repelling force is generated (Fig. 2), the reference is taken as the initial location of the dust particle. It should be noted that the x-axis is located along the electrode length, y-axis is normal to the plate surface and z-axis is in the plane of the plate surface and normal to the x-axis. Moreover, the zooming of the high speed camera onto the dust particle causes the image clarity loss and even disappearing during the optical tracking; therefore, the maximum zooming with sufficient image clarity is selected to trace the particle trajectory. The red circles are used in the high speed camera image to enable the location of the dust particle. It is evident from Fig. 10 that the dust particles follows 3-dimensional trajectory; however, the distance covered by the dust particle is longer along the x-axis than those corresponding to the displacements in the y, and z-axes. It should be noted that the y-axis is normal to the plate surface where the electrostatic electrode is located. The experimental data for the distance covered by the dust particles along the x, y, and z-axes with time are also shown in Fig. 10 for comparison. The dust particle has higher velocities along the y-axis as compared to those corresponding to other axes, which indicates that the impulsive force is the largest along the y-axis. However, the dust particle velocity remains relatively larger along the x-axis as compared to that of the z-axis. The attainment of the large velocity along the x-axis is attributed to the presence of the repelling forces occurring due to the electrostatic charge along the x-axis (electrode axis direction). The velocity of the dust particles increases sharply under the influence of the electrostatic repulsion force and as the time progresses the particle velocity reduces gradually. As time time progresses further, the velocity reduces reaching almost zero under the influence of the gravitational potential energy. The zero velocity along the x-axis corresponds to the maximum distance covered by the particle. The time occurrence of the zero velocity along the y-axis, where the particle displacement is the maximum in the vertical direction normal to the surface, slightly varies along the x and z-axes. In this case, the occurrence of zero velocity along the x and z-axes becomes slightly longer than that of the y-axis. This indicates that the particle continues to move in the x, and z-axes while the particle reaches its peak location along the y-axis. Figure 11 shows the particle acceleration along the x, y and z-axes. The particle acceleration remains high in the early period due to the particle movement from the near region of the surface under the repelling electrostatic force. As the time progresses, the particle acceleration along the y-axis reduces and it reduces gradually up to the point when the velocity reaches zero. The particle accelerates under the influence of the gravitational pulling force from the point of zero velocity towards the surface during the falling cycle. In this case, the acceleration of the particle remains slightly lower in the falling cycle as compared to the repelling cycle. Therefore, the acceleration due to the repelling electrostatic force is considerably higher than the gravitational pull, i.e. initial acceleration over comes the gravitational pull, pinning force due to particle adhesion, drag force due to air resistance, and forces generated due to repelled particles interactions.

To determine the particle acceleration by the electrostatic force during repelling from the surface, the force balance for the repelling particle is considered. The only external force repelling the particle from the surface is the electrostatic force, which is generated by the high voltage unit. The electrostatic force (*F*_*Els*_), which is generated by the electrostatic charge can be expressed as:*F*_*Els*_ = *QE*, where *Q* is the electrostatic charge on the particle to be repelled and *E* corresponds to the electric field strength applied on the particle. The electrostatic field strength is associated with the applied voltage and the distance between the high voltage electrode and the dust particle. The electrostatic charge on the dust particle depends on the constituting elements of the dust particle; in which case, the charge may fluctuate depending on the dust particles composition^32^. However, in the present analysis, the average electrostatic charge is considered on the particle. The electrostatic charge can be formulated through the Gauss’s law^33^, which yields:$\,Q=EA{\varepsilon }_{p}$, here, *A* is the area of the dust particle and ε_p_ is the electrostatic permittivity of the material used in the plate, where the dust particle is resting on, prior to repelling. After assuming the area of the dust particles can be presented as $A=f\pi {R}_{p}^{2}$, here *f* is the correction factor and changes within 0.3 ≤ f ≤ 0.6 because of the irregularity of the dust particle geometric feature, and *R*_*p*_ is the dust particle equivalent radius in relation to the equivalent spherical shape. After inserting the electrostatic charge and the area in the electrostatic force formulation, it yields:

1 $${F}_{Els}={E}^{2}f\pi {R}_{p}^{2}{\varepsilon }_{p}$$

**Figure 12:**
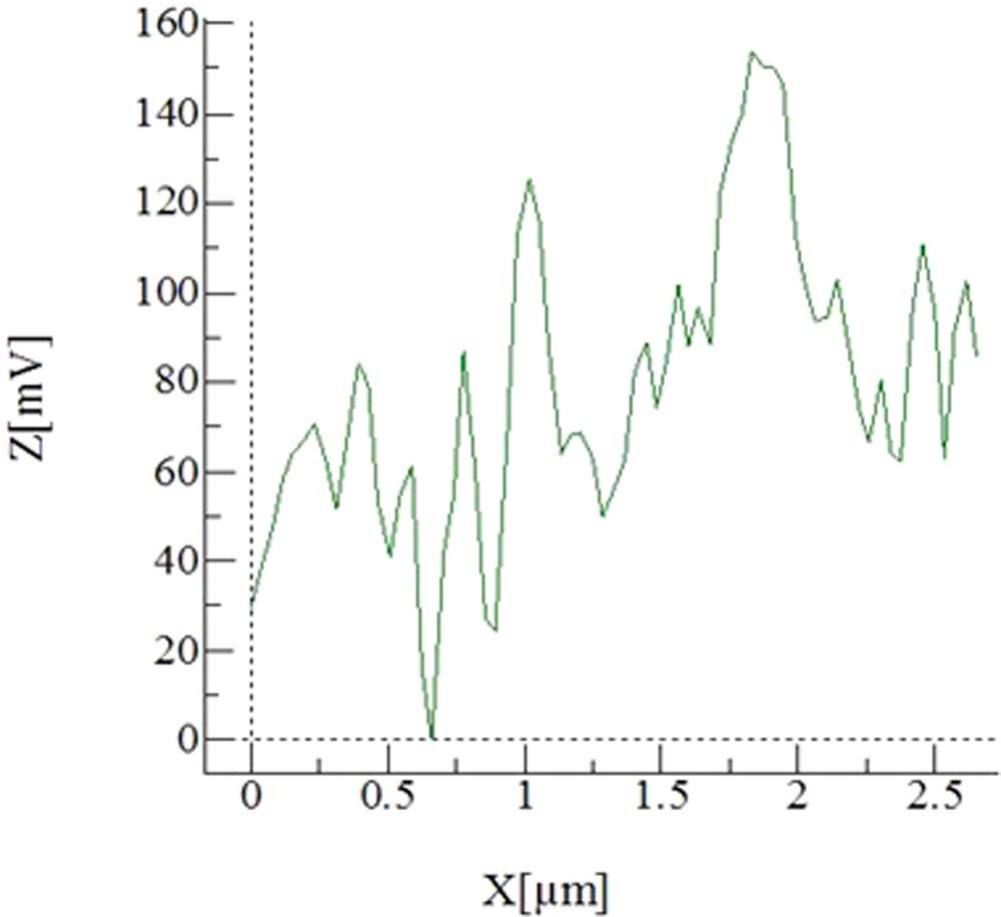
AFM tip response in friction mode during the dust particle removal from surface.

However, the electrostatic force overcomes the adhesion and gravitational forces of the dust particle on the plate surface prior to the dust particle repelling. In addition, the electrostatic force remains influential on the dust particles after repelling from the surface. In this case, the electrostatic field (${F}_{Els} \sim \frac{{\rm{\Delta }}V}{{\rm{\Delta }}h}$, here ΔV corresponds the applied voltage and *h* is spacing between the copper electrode, where the electric potential is applied, and the dust particle) is reduced because of the increasing dust particles elevation in the air ambient. The additional electrostatic force depends on the dust particle inflight location in air (height) because of the height dependence of the electrostatic field. The additional electrostatic force can be formulated as ${F}_{(Els)addition}=\,E{(h)}^{2}f\pi {R}_{dp}^{2}{\varepsilon }_{air}$, here *E*(*h*) represents the height dependent electrostatic field influencing the repelled dust particle during inflight condition. It should be noted that the high voltage is applied within a single square pulse form with the pulse length of 0.4 s. Therefore, the electrostatic force remains active during the pulse period. However, the electrostatic influence among the repelled dust particles in air also contributes to the particle motion (inflight condition) in air. The dust particles compose of various elements and the electrostatic charge on each dust particle may vary, which in turn, can generate electrostatic force in between the repelled dust particles in air. The electrostatic force between the dust particles can be formulate through ${F}_{B(Els)}=\frac{K{Q}_{1}{Q}_{2}}{{l}^{2}}$, where *K* is the electrostatic constant, *Q*_1_ and *Q*_2_ is the charge amounts of dust particles, and *l* is the distance between the dust particles. It is considered that the charge amount of the dust particles with small sizes remains small and the spacing in between the dust particles are large in air; consequently, the electrostatic influence, due to charge differences, among the dust particles in air is omitted, i.e., it is considered that the repelled dust particle trajectory is not influenced by the other repelled dust particle in air. Moreover, the van der Waals force is the main contributor to the adhesion force in dry and elctrodynamically neutral environment^34^. Few models were developed formulating the adhesion force between the surfaces and the particles^35–38^. Since the dust particle surface is rough, the model improved by Rabinovich *et al*.^38^ can be used to formulate the dust particle adhesion on the plate surface. The adhesion force between the dust particle and the plate surface takes the form ${F}_{Ad}=\frac{A{R}_{pd}}{12{Z}_{0}^{2}}(\frac{1}{1+\frac{{R}_{pd}}{1.48{r}_{s}}}+\frac{1}{{(1+\frac{1.48{r}_{s}}{{Z}_{0}})}^{2}})$, here *A* represents the Hamaker constant (A = 0.48 × 10^−20^ J for SiO_2,_^39^) and *Z*_*o*_ is the particle spacing, which is same order of separation distance between the particle surface and the flat surface, *ε* is the surface roughness of the flat surface, and *r*_*s*_ is the roughness parameter of the dust particle surface. The surface roughness of the plate is in the order of 120 nm and the roughness parameter of the dust particle is estimated roughly from the SEM micrographs as *r* = 0.62. On the other hand, in order to assess the adhesion of the dust particle on the plate surface, atomic force microscopy measurement of the adhesion of the dust particle is carried out. The deflection of the atomic force microscopy tip can be related to the adhesion force of the dust particle on the plate surface^22^. In this case, the force of adhesion (*F*_*add*_) can be written in the form of $\,{F}_{add}=k\sigma {\rm{\Delta }}V$^22^, here *k* represents the spring constant of the cantilever tip (N/m) *σ* is the slope of the displacement of the tip over the resulting probe voltage (*Δz*/*ΔV*, m/V), and *ΔV* is the probe voltage as a result of the atomic force microscope tip scanning on the surface in the contact mode. Figure 12 depicts the atomic force microscope tip response, in terms of mV, when about 10 µm size dust particle is moved on plate surface. The tangential force required to move the dust particle on the plate surface is similar order of the adhesion force. The maximum peak corresponds to the tangential force and the small peaks are related to the frictional force on the surface, i.e. the maximum peak corresponds to the initiation of the dust movement on the flat surface. In the measurements, the following data is adopted from the calibration of the atomic force microscopy tip; *kσ* = 5.80275 × 10^−13^ N/mV. Moreover, incorporating the deflection relation for the atomic microscope tip, the adhesion force can be determined. Hence, the adhesion force is determined in the order of 8.12844 × 10^−11^ N for about 10 µm size dust particle located on the hydrophilic glass surface. However, using the relation introduced by Rabinovich *et al*.^38^ for the adhesion force, the adhesion force is determined as 4.3 × 10^−11^ N for 10 µm size dust particle. Consequently, the adhesion force measured and calculated are almost similar order. The tangential force measurement is repeated for the dust particles of about 10 µm size located on the hydrophobic surface. The adhesion force measured for the dust particle is 2.23417 × 10^−11^ N on the hydrophobic surface. Hence, the adhesion of the dust particle on the surface is reduced by almost 3.6 times as the surface becomes hydrophobic. Moreover, adhesion force measurement is extended to include the small dust particle, which is about 1.1 µm size, located on both the hydrophilic and the hydrophobic surfaces. The adhesion force measured for 1.1 µm size particle located on the hydrophilic surface is about 2.52711 × 10^−10^ N while it is 6.2217 × 10^−11^ N as the particle located on the hydrophobic surface. The adhesion force increases almost 3 times for the hydrophilic surface as the small particle is located on the hydrophilic surface. However, the adhesion force increases almost 3 times as the small particle is located on the hydrophobic surface. Consequently, reducing the dust particle size increases the adhesion force on both hydrophobic and hydrophilic surfaces. The probable explanation of this behavior is due to: (i) the contact area at the dust surface interface, which becomes smoother as the dust particle size reduces, and (ii) the charge forces between the particle and the surface. However, further research studies are needed to explore the influence of the dust particle size on the adhesion force; hence, the extensive investigation is left for the future study. In order to ensure the repeatability of the measurements, the tests are repeated nine times. And the error estimated, based on the repeatability of the experiments, is in the order of 9% for the adhesion force measurements. Moreover, the drag force is one of forces retarding the repelling dust particle in air. The drag force of the particle can be written in terms of the dust particle velocity in the form of ${F}_{D}=\frac{1}{2}{C}_{D}\,\rho \,{v}^{2}\,{A}_{x}$, where *C*_*D*_ is the drag coefficient, *v* is the dust particle velocity, *ρ* is the density of air, and *A*_*x*_ is the dust particle cross-sectional area. The drag coefficient is related to the Reynolds number and incorporating the Reynolds number, it reduces to: ${F}_{D}=\,3\,\pi \,\mu \,v\,{D}_{p}$, where *D*_*p*_ is the diameter of the dust particle and µ is the dynamic viscosity of air. Incorporating the data, the Reynolds number for about 10 µm size of the dust particle with inflight velocity in the range of 0.0.011 m/s is in the order of 2.32 × 10^−3^, which is incorporated in the Reynolds number calculation. Consequently, according to the Stokes theorem, the drag coefficient takes the form C_D_ = 24/Re^40^. Although the drag force depends on the dust particle velocity, the maximum drag force is in the order of 3.29241 × 10^−11^ N. Consequently, it remains less than the electrostatic and adhesion forces. The force balance for the dust particle in air, which is just repelled from the surface, is:

2 $${F}_{i}=({F}_{Els}-{F}_{add})+{F}_{(Els)Addition}-{F}_{w}-{F}_{D}$$

where *F*_*i*_ is the resulting inertia force of the dust particle, *F*_*Els*_ is the electrostatic force initially imposed onto the dust particles, *F*_*(Els)Addition*_ is the additional electrostatic force acting on the dust particle in air (inflight condition) during the period of applied voltage pulse, *F*_*add*_ is the adhesion force of the dust particle on the flat plate, *F*_*w*_ is the gravitational force (weight) acting on the dust particle, and *F*_*D*_ is the drag force. It should be noted that *F*_*Els*_ and *F*_*add*_ forces are not the active forces onset of the dust repelling, i.e. once the dust particle repels from the surface their effect ceases. In terms of the dust particle acceleration, Eq. 2 can be rearranged, i.e.:

3 $${a}_{p}=\frac{1}{{m}_{p}}({F}_{Els}-{F}_{add})+\frac{1}{{m}_{p}}{E}^{2}\{h\}f\pi {R}_{p}^{2}{\varepsilon }_{air}-g-\frac{3}{{m}_{p}}\,\pi \,\mu \,v\,{D}_{p}$$

here *m*_*p*_ is the dust particle mass, *D*_*p*_ is the dust particle diameter, and *h* is the dust height during inflight, and {} resembles the functional relation, which is assumed to be quadratic. The initial velocity of the dust particle can be estimated from the impulse relation after considering initial impulse on the dust particle due to electrostatic impulse. It yields: $Vo=\frac{({F}_{Els}-{F}_{add}-{F}_{w})}{m}{\rm{\Delta }}t$, here, Δt is selected as 15 ms, when the dust particles are recorded in the surface region in air. In addition, the high voltage pulse reaches at peak almost after 15 ms. The initial velocity of the dust particle (*v*_*o*_) is estimated as 0.01594 m/s. The velocity of the inflight particle reaching the maximum height above the plate surface is:

4 $$v(t)={v}_{o}t-[\frac{1}{{m}_{p}}({F}_{Els}-{F}_{add})+\frac{1}{{m}_{p}}{E}^{2}\{h\}f\pi {R}_{p}^{2}{\varepsilon }_{air}-g-\frac{3}{{m}_{p}}\,\pi \,\mu \,v\,{D}_{p}]{t}^{2}$$

**Figure 13:**
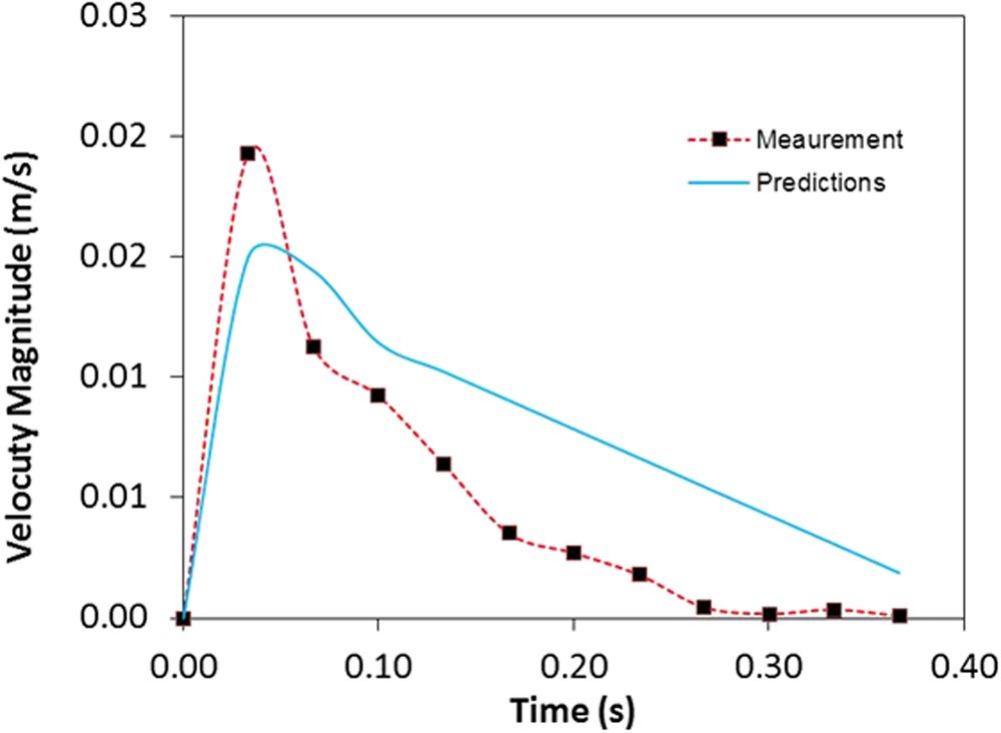
Velocity magnitude predicted and obtained from the measurements.

**Figure 14:**
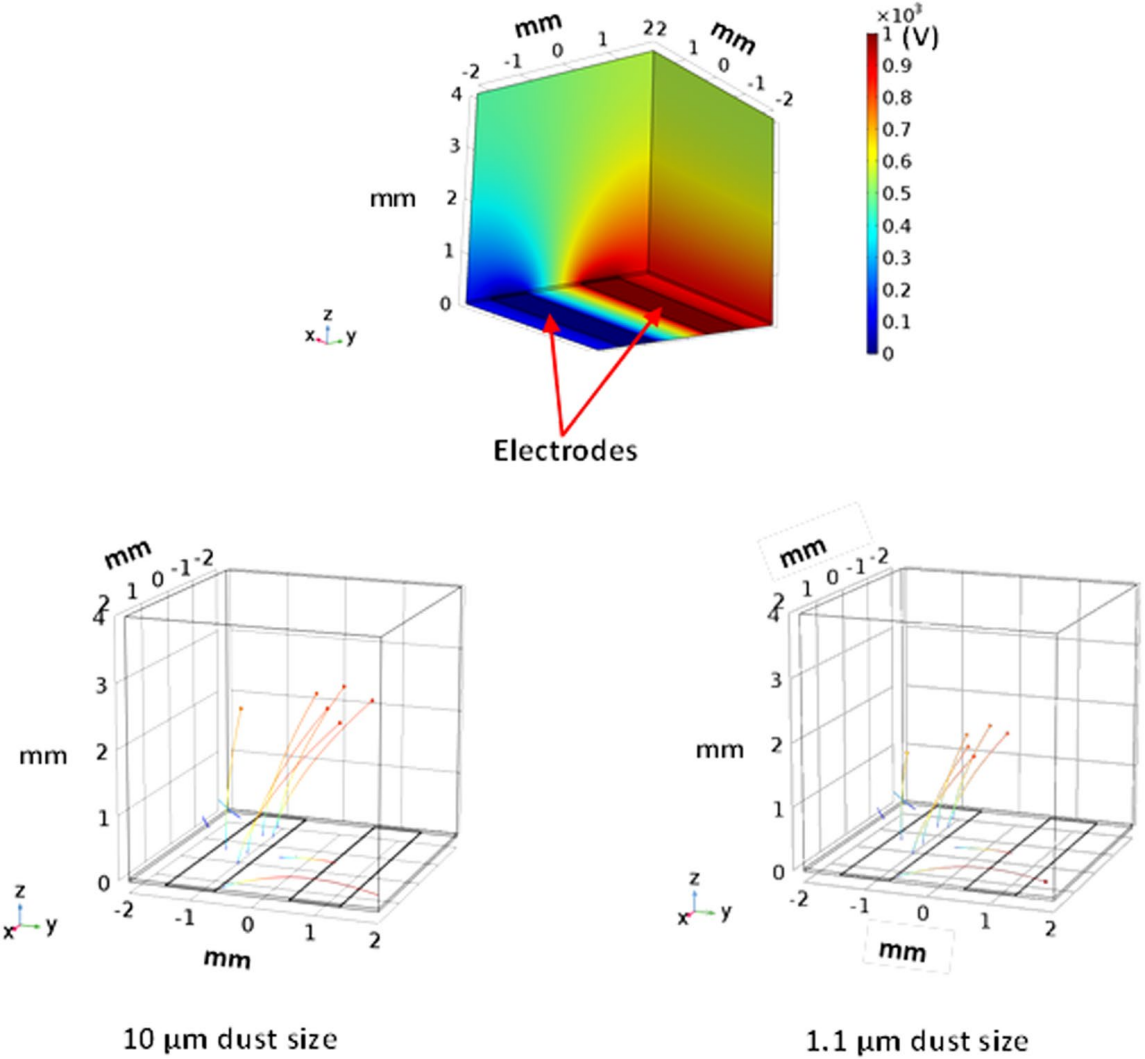
Electrostatic potential and repelling dust particle trajectories: (**a**) electrostatic potential, (**b**) trajectories of 10 µm dust particles, and (**c**) trajectories of 1.1 µm dust particles.

Equation 4 is used to predict the velocity of the repelled particle form the plate surface. Figure 13 shows the velocity magnitude of the dust particle predicted from the analytical formulation (Eq. 4) and obtained from the measurements. It should be noted that the velocity of the repelled dust particle is obtained from the high speed camera data along the x, y and z-axes. Therefore, the velocity magnitude ($=\sqrt{{v}_{x}^{2}+{v}_{y}^{2}+{v}_{z}^{2}}$, where *v*_*x*_, *v*_*y*_, *v*_*z*_ are the repelled particle velocity components in *x*, *y*, and *z*-axes, respectively) is presented in Fig. 13 resembling the measured velocity from the high speed camera. The prediction from the analytical formulation (Eq. 4) almost agrees with the velocity data obtained from the experiment. The differences in both results are associated with experimental errors and the assumptions made in the analytical formulations, such as correction factor introduced for the geometric abnormalities of the dust particle. Moreover, electrostatic repelling of dust from the plate surface is numerically simulated using COMSOL Multiphysics code^40^ while incorporating the flat configuration and the conditions used in the experiments. The details of the formulation are given in Supplementary Material S2. Figure (14a) shows the 3-dimensional simulation results for volumetric voltage distribution while Fig. (14b,c) show the particle trajectories resembling the dust in 3-dimensional domain. The dust particle shows similar behavior above the plate surface; in which case, the maximum vertical heights of the dust particles of 10 µm size are within the range of 2.5 mm, which are inconsistent with the measurements. The simulations are extended incorporating the small size dust particles. The dust particles size is selected as 1.1 µm and the adhesion force for the dust particle is considered as the opposing to the repelling force. The trajectory of the dust particles with 1.1 µm size is shown in Fig. (14c). The maximum vertical height of the dust particles remains smaller than the large particles (Fig. 14b) because of the relatively larger adhesion force (2.52711 × 10^−10^ N) for the small dust particles than the large size particles (8.12844 × 10^−11^ N) on the hydrophilic glass.

**Figure 15:**
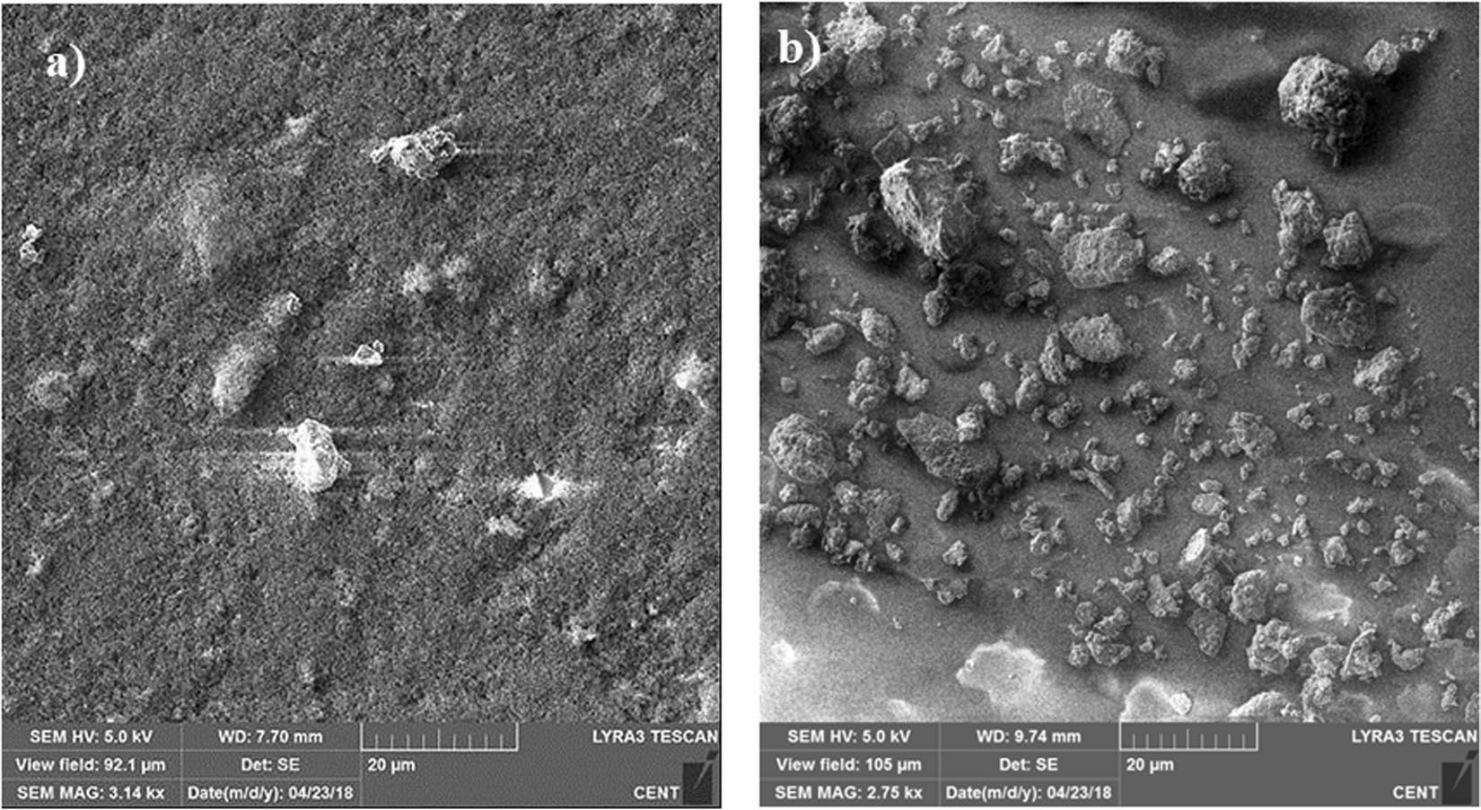
SEM micrographs of dust particles residues on the plate surface: (**a**) hydrophobic surface, and (**b**) hydrophilic surface.

Figure 15 shows SEM micrographs of the dust residues on the surface after the electrostatic repelling from the hydrophobic and hydrophilic plate surfaces. The micrographs are obtained for the residues of the dust particles located along the electrostatic wire (*x*-axis direction). In general, small size dust particles are remained on the hydrophobic surface after applying the electrostatic repelling force (Fig. 15a). This is associated with one or all of the findings: (i) small size dust particles has small mass; in which case, the repelling electrostatic force does not overcome particularly the pinning force due to adhesion, (ii) although some small dust particles are attach on the large size particle surfaces (Fig. 7b), some of the small particles may attach on the plate surface upon deposition along the wire; in which case, pinning force increases significantly because of the strong adhesion between the small size dust particles and the surface. The elemental composition of the residues of the small dust particles is assessed using the energy dispersive spectroscopy. Table 1 provides the elemental composition of the residues of the dust particles. The elemental composition changes in the dust residues, particularly for the small size dust particles (≤0.8 µm). The oxygen content in the dust particle is higher for the small dust particles residues than the average and large size dust particles. This indicates that the small dust dust particles have lower density than that of the average size dust particles (2800 kg/m^3^). The density of the small dust particles residues is estimated in the order of 1600 kg/m^3^. In order to assess the adhesion of the small and average size dust particles on the plate surface, the tangential force required removing the dust particles from the surface is measured using the atomic force microscopy with its tip operating on the friction mode. In this case, on the hydrophobic surface, the adhesion force obtained from the atomic force microscopy data for the small dust particles (∼0.8 µm) is in the order of 5.1242 × 10^−11^ N, which is almost twice larger than that of average dust particle (2.5711 × 10^−10^ N for about 1.1 µm size dust particle). It should be noted that the average density of the small dust particle is in the order of 1600 kg/m^3^ and the dust size of the dust particles used in the measurement is in the order of 0.6 μm–0.8 μm. In this case, the low density small particles remain on the surface when the electrostatic repelling force is applied. Moreover, the dust particle repelling experiments are repeated incorporating the normal glasses; in which case, the dust particles are deposited on the glass surface along the copper electrode line and same conditions of electronic circuit setting, which are used for the functionalized silica particles deposited glass surface, are adopted. The SEM micrograph of the residues of the dust particles on the glass surfaces, after introducing the electrostatic force, is shown in Fig. 15b. The concentration of the dust particles on the glass surface remains significantly higher than that corresponding to the functionalized silica particles deposited surface (Fig. 15a). In order to assess the adhesion of the dust particles on the plain glass surface, the atomic force microscopy measurements for the tangential force required removing the dust particles from the surface are repeated for the plain glass surface. The measurements reveal that the adhesion force of the dust particles with the size of about 10 µm on the plain glass surface is in the order of 8.12844 × 10^−11^ N, which is almost 3 times more than that corresponds to the functionalized silica particles deposited surface for almost same size particle (2.23417 × 10^−11^ N). Consequently, hydrophobic wetting state created by the deposition of the functionalized silica particles significantly lowers the dust particles adhesion on the surface.

## Conclusion

One of the methods removing the dust particles from the surfaces is utilizing the electrostatic forces towards repelling the dust particles. Consequently, influence of the electrostatic force on the dust particles repelling from the flat surfaces is investigated. The dust particles are collected from the local area of Dammam in the Kingdom of Saudi Arabia and elemental composition, size distribution, and shapes of the dust particles are analyzed using the analytical tools. The effect of the wetting state of the flat surface on the dust repelling is considered and functionalized silica particles are deposited onto the flat surface achieving the hydrophobic wetting state on the surface. High speed camera is used to monitor the dynamics of the dust particles repelling form the hydrophobic and hydrophilic surfaces. The dynamics of the dust particles is examined analytically incorporating the force balance; hence, the velocity of the dust particles repelling is formulated. The particle velocity predicted from the analytical formulation is compared to those obtained from the high speed camera data. The findings reveal that deposition of the functionalized silica particles alters the wetting state of the surface from hydrophilic to hydrophobic state; in which case, uniform hydrophobic wetting state is achieved across the surface with droplet contact angle of 158° ± 2° and contact angle hysteresis of 2° ± 1°. The dust particles have various shapes having the average size of in the order of 1.2 µm. The dust particles compose of various elements; however, oxygen content is high in the small size dust particles, which lowers the dust particle mass density from 2800 kg/m^3^ to 1600 kg/m^3^. Introducing the electrostatic effect, via high voltage discharge in the pulse form, results in the dust particles repelling from the plate surface. The repelled dust particles follow three-dimensional trajectory in air under the electrostatic impulse, drag, and gravitational forces. The dust particle inflight velocity remains largest along the y-axis, which is perpendicular to the plate surface. The analytical prediction of the dust particle (∼10 µm) velocity agrees with that obtained from the high speed camera data. Some small size particles (0.8 µm≤) remain on the hydrophobic plate surface after the electrostatic impulse pulse is introduced. This behavior is attributed to: (i) small size particles, in general, have large oxygen content and their density is lower than those of the average size particles; in which case, inertia force accelerating the particle remains low, (ii) adhesion force, as measured by the atomic force microscopy, is higher for the small size dust particles as compared to that of the average size particles. Nevertheless, using the hydrophobic surface lowers the dust residues on the surface after repelling of the dust particles via electrostatic force. The adhesion force of the dust particles becomes larger for the hydrophilic plain glass surfaces as compared to that corresponding to the hydrophobic surface. Consequently, using the hydrophobic plate surface lowers the dust particles residues on the surface after electrostatic repelling. The present study gives insight into the dust particles repelling from hydrophilic and hydrophobic surfaces under the electrostatic influence. It provides useful information about the environmental dust particles and dust removal from the surfaces.

## Supplementary information

Droplet Method for Formulation of Surface Free Energy

Dimensional formulation of Electrostatic Influence
