# Supplementary material for: Environmental Dust Particles Repelling from A Hydrophobic Surface under Electrostatic Influence
Source: Sci Rep. 2019 Jun 18;9:8703. doi: 10.1038/s41598-019-44992-9 (PMC6582155; doi:10.1038/s41598-019-44992-9)
Supplement: Supplementary file 1 — Droplet Method for Formulation of Surface Free Energy [file 41598_2019_44992_MOESM1_ESM.pdf]

Supplementary Information

ENVIRONMENTAL DUST PARTICLES REPELLING FROM A HYDROPHOBIC SURFACE UNDER ELECTROSTATIC INFLUENCE

B.S. Yilbas*[,1,2], Hussain Al-Qahtani[1], Abdullah Al-Sharafi[1], Saeed Bahattab[1], Ghassan Hassan[1,2], N. Al-Aqeeli[1], M. Kassas[3]

[1]Mechanical Engineering Department, King Fahd University of Petroleum and Minerals, Saudi Arabia

[2]Center of Excellence in Renewable Energy, King Fahd University of Petroleum & Minerals, Dhahran, Saudi Arabia

[3]Electrical Engineering Department, King Fahd University of Petroleum & Minerals, Dhahran, Saudi Arabia

*Corresponding author. Email: bsyilbas@kfupm.edu.sa; Phone: +966 3 860 4481

Droplet Method for Formulation of Surface Free Energy

The assessment of the surface free energy of solids and liquids was introduced earlier by van Oss et al.[1]. The relation for the surface free energy can be written as:

$$\gamma = \gamma^{L} + \gamma^{P} \tag{1}$$

where γ^{L} is the apolar component due to Lifshitz-van der Waals intermolecular interactions and γ^{P} is attributed to electron-acceptor and electron donor intermolecular interactions. The apolar component γ^{P} owing to electron-acceptor and electron donor intermolecular interactions can be expressed as[1, 2].

$$\gamma^{P} = 2\sqrt{\gamma^{+}.\gamma^{-}} \tag{2}$$

where γ^{+} and γ^{-} are the electron acceptor and electron donor parameters of the acid-base component of the solid and liquid surface free energy, respectively. The interfacial free energy for a solid-liquid system can be expressed as[1, 2]:

$$\gamma_{SL} = \gamma_{S} + \gamma_{L} - 2\sqrt{\gamma_{S}^{L}.\gamma_{L}^{L}} - 2\sqrt{\gamma_{S}^{+}.\gamma_{L}^{-}} - 2\sqrt{\gamma_{S}^{-}.\gamma_{L}^{+}} \tag{3}$$

where subscripts *S* and *L* represent solid and liquid phases, respectively. The Young's equation for the surface free energy of a solid becomes[2]:

$$\gamma_{L} \cos\theta = \gamma_{S} - \gamma_{SL} - Pe_{L} \tag{4}$$

where γ_{S} represents the solid surface free energy, γ_{SL} corresponds to the interfacial solid-liquid free energy, γ_{L} is the liquid surface tension, θ is the droplet contact angle, and Pe_{L} is the pressure of the liquid film, which is negligibly small and considered to be zero[1]. Combining Eqs. 3 and 4 and re-arranging them yield:

$$\gamma_L(cos\theta + 1) = 2\sqrt{\gamma_S^L.\gamma_L^L} + 2\sqrt{\gamma_S^+.\gamma_L^-} + 2\sqrt{\gamma_S^-.\gamma_L^+} \quad (5)$$

Eq. 5 can be incorporated towards evaluation of γ_S^L, γ_S^+, and γ_S^- while using the contact angle data and γ_L^L, γ_L^+, and γ_L^-. The data for γ_L^L, γ_L^+, and γ_L^- can be found from the literature for water, glycerol, and diiodomethane, which are given in Table 2[1, 2, 3]. The contact angle measurements were repeated five times to ensure the measurement repeatability of the data in line with the previous study[4].

REFERENCES

1 Van Oss, C. J., Chaudhury, M. K. & Good, R. J. Interfacial Lifshitz-van der Waals and polar interactions in macroscopic systems. *Chemical Reviews* **88**, 927-941 (1988).

2 Oss, C. v., Good, R. J. & Busscher, R. Estimation of the polar surface tension parameters of glycerol and formamide, for use in contact angle measurements on polar solids. *Journal of Dispersion Science and Technology* **11**, 75-81 (1990).

3 Sun, C.-C. *et al.* Surface free energy of alloy nitride coatings deposited using closed field unbalanced magnetron sputter ion plating. *Materials transactions* **47**, 2533-2539 (2006).

4 Heib, F. *et al.* High-precision drop shape analysis (HPDSA) of quasistatic contact angles on silanized silicon wafers with different surface topographies during inclining-plate measurements: Influence of the surface roughness on the contact line dynamics. *Applied Surface Science* **342**, 11-25 (2015).
