# Supplementary material for: Environmental Dust Particles Repelling from A Hydrophobic Surface under Electrostatic Influence
Source: Sci Rep. 2019 Jun 18;9:8703. doi: 10.1038/s41598-019-44992-9 (PMC6582155; doi:10.1038/s41598-019-44992-9)
Supplement: Supplementary file 2 — Dimensional formulation of Electrostatic Influence [file 41598_2019_44992_MOESM2_ESM.pdf]

Supplementary Information

ENVIRONMENTAL DUST PARTICLES REPELLING FROM A HYDROPHOBIC SURFACE UNDER ELECTROSTATIC INFLUENCE

B.S. Yilbas*[,1,2], Hussain Al-Qahtani[1], Abdullah Al-Sharafi[1], Saeed Bahattab[1], Ghassan Hassan[1,2], N. Al-Aqeeli[1], M. Kassas[3]

[1]Mechanical Engineering Department, King Fahd University of Petroleum and Minerals, Saudi Arabia

[2]Center of Excellence in Renewable Energy, King Fahd University of Petroleum & Minerals, Dhahran, Saudi Arabia

[3]Electrical Engineering Department, King Fahd University of Petroleum & Minerals, Dhahran, Saudi Arabia

*Corresponding author. Email: bsyilbas@kfupm.edu.sa; Phone: +966 3 860 4481

Dimensional formulation of Electrostatic Influence

The Dielectrophoretic force feature adds the following contribution to F_{ext} for the case of a static electric field[1]:

$$F_{ext} = 2\pi r_p^3 \varepsilon_o \varepsilon_f \left(\frac{\varepsilon_p - \varepsilon_f}{\varepsilon_p - 2\varepsilon_f}\right) \nabla |E|^2 \quad (1)$$

Where r_d is the particle radius, ε_f is the relative permittivity of the fluid, ε_p is the relative permittivity of the particle, and **E** is the electric field. In the case that the electric field is computed in the frequency domain, the following is added:

$$F_{ext} = 2\pi r_p^3 \varepsilon_o real(\varepsilon_f) real\left(\frac{\varepsilon_p - \varepsilon_f}{\varepsilon_p - 2\varepsilon_f}\right) \nabla |E_{rms}|^2 \quad (2)$$

where E_{rms} denotes the root mean square electric field.

COMSOL Multiphysics code[2] is used to simulate electrostatic influence on the dust particle.

REFERENCES

1 Bruus, H. *Theoretical microfluidics*. Vol. 18 (Oxford university press, Oxford, 2008).

2 http://www.comsol.com/comsol-multiphysics, 2019.
